# The feasibility of discriminating BRONJ lesion bone with Raman spectroscopy

**DOI:** 10.3389/fendo.2023.1099889

**Published:** 2023-05-08

**Authors:** Chengwan Xia, Yumei Pu, Qian Zhang, Qingang Hu, Yuxin Wang

**Affiliations:** Department of Oral and Maxillofacial Trauma Orthognathic Plastic Surgery, Nanjing Stomatological Hospital, Medical School of Nanjing University, Nanjing, China

**Keywords:** bisphosphonate-related osteonecrosis of the jaw, Raman spectral, lesion bone, bisphosphonates, zoledronate

## Abstract

**Background:**

With the frequent use of Bisphosphonates (BPs), the morbidity of BP-related osteonecrosis of the jaw (BRONJ) is also increasing. However, the prevention and treatment of BRONJ is faced with enormous challenges. This study aimed to illuminate the influence of BP administration in the rat mandible and explore the feasibility of discriminating BRONJ lesion bone with Raman spectroscopy.

**Materials and methods:**

First, we explored the time- and mode-dependent effects of BP administration on the rat mandible with Raman spectroscopy. Second, the BRONJ rat model was constructed, and the lesion and healthy bone components were analyzed using Raman spectroscopy.

**Results:**

When only BPs were administered, no rats showed BRONJ symptoms, and no difference could be found in the Raman spectra. However, when combined with local surgery, six (6/8) rats showed BRONJ symptoms. The Raman spectra also showed a significant difference between the lesion and healthy bone.

**Conclusion:**

In the progression of BRONJ, BPs and local stimulation play an essential role. Both BPs administration and local stimulation need to be controlled to prevent BRONJ. Moreover, BRONJ lesion bone in rats could be discriminated with Raman spectroscopy. This novel method would become a complement in the treatment of BRONJ in the future.

## 1 Introduction

Bisphosphonates (BPs), that act as antiresorptive drugs, have been widely used in the prevention and treatment of osteoporosis, osteolysis associated with bone metastases, Paget’s disease of bone, and hypercalcemia (1–5). The morbidity of bisphosphonate-related osteonecrosis of the jaw (BRONJ) complications also increased because of the frequent administration of BPs (6). BRONJ can cause pain, tooth mobility, halitosis, paresthesia, bone sequestrum formation, and intra-oral or extra-oral fistula, greatly affecting the patient quality of life (7). The unclear pathogenesis of BRONJ still hinders its prevention and treatment.

McDonald et al. (8) hold the view that osteoclasts can take up BPs deposited in bone, which leads to the inactivation and retraction of the ruflled membrane, hence breaking the balance of bone metabolism. Walter et al. (9) showed that BPs could inhibit the proliferation, differentiation, adhesion, and migration of endothelial cells, induce programmed death of vascular endothelial cells, and lead to the inhibition of angiogenesis. After observing the pathological section of necrotic bone tissue, Boff et al. (10) found that many bacterial toxins and inflammatory factors released by actinomycetes could induce programmed osteoclast death, which results in the formation of necrotic bone. Some researchers found that BPs are more likely to be released and activated in an acidic environment, and are more cytotoxic, which reveals the promoting effect of infection and inflammation in the BRONJ formation (2). Despite these findings, the pathogenesis of BRONJ has not been fully established.

Currently, BRONJ treatment is still controversial. Although conservative treatment could slow down the progression of BRONJ and relieve the patient’s clinical symptoms, surgical removal of necrotic bone still needs to be considered in all BRONJ stages (11). Moreover, because of the difficulty of determining the extent of the affected bone, too much bone tissue may be removed during surgery, leading to unnecessary complications or rapid recurrence. Therefore, it would be of great significance for BRONJ patients if a novel way can be established to accurately determine the extent of lesion bone.

Many methods have been used to evaluate bone tissue structure and components, such as micro-CT, X-ray diffraction, and infrared spectroscopy. However, all of these methods have their limitations (12). Raman spectroscopy (a nondestructive measurement method) has been widely used in many fields, such as materials science, chemistry, physics, biology, and medicine. Compared with previous methods, Raman spectroscopy has its unique advantages: 1) as Raman is a scattering phenomenon, it can be used in reflection mode on solid samples; 2) in contrast to Fourier-transform infrared spectroscopy (FTIR), this technique is relatively insensitive to water, allowing for the analysis of fully hydrated samples; 3) as a nondestructive technique, the same sample can be examined using other methods later; and 4) it can simultaneously detect organic and mineral phases (12). Because of these unique advantages, various research studies have focused on using Raman spectroscopy to detect biological samples like tumors and bone tissues (13–15). However, the use of Raman spectroscopy to discriminate BRONJ has rarely been reported.

Therefore, this study aimed to analyze the structure and composition of the mandible bone tissue before and after BP administration and further explore the feasibility of discriminating the BRONJ lesion bone with Raman spectroscopy.

## 2 Materials and methods

### 2.1 Materials and animals

Zoledronate (ZA) was purchased from Aosaikang Pharmaceutical Co., Ltd (JiangSu, China). The Sprague Dawley (SD) rats (female, 4–6 weeks) were brought from the Comparative Medicine Centre of Yangzhou University. All animal procedures were performed in accordance with the Guidelines for the Care and Use of Laboratory Animals of Nanjing University, and the Animal Ethics Committee of the Institute Affiliated Stomatology Hospital, Nanjing University Medical School approved the experiments. All animal procedures were performed in accordance with the Guidelines for Care and Use of Laboratory Animals of Nanjing University. The rats were acclimated for 1 week to the local environment (24, 12h/12h light dark cycle).

### 2.2 Time-dependent effects of BP administration on the rat mandible

**Figure 1:**
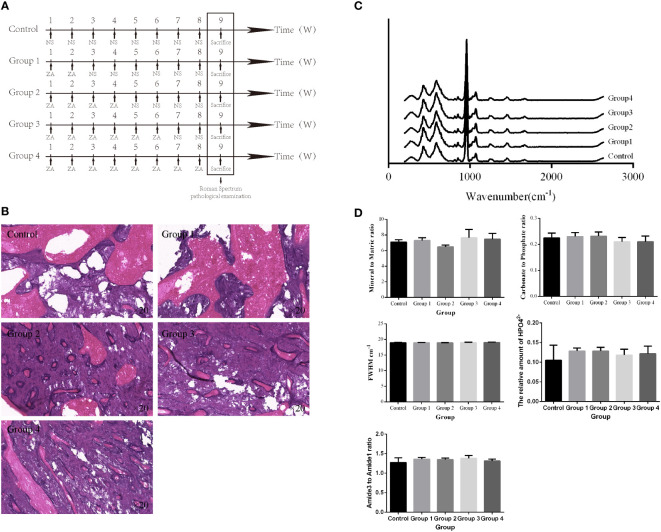
Time-dependent effects of BP administration on the rat mandible. **(A)** Schedule of BP administration to rats. **(B)** Pathology images show increased trabeculae with increased BP administration times in the cancellous bone region. **(C, D)** The Raman spectra from different groups and the derived parameters show no significant difference in the mandibular components and structure.

Thirty (16) rats were prospectively and randomly divided into 5 groups. All rats were injected with a dose of ZA (100 μg/kg) (17) or an equivalent dose of normal saline (NS) through the tail vein once a week for 8 weeks (**Figure 1A**). On week 9, all rats were sacrificed, and their mandibles were dissected for Raman spectroscopy and pathological examination.

### 2.3 Mode-dependent effects of BP administration on the rat mandible

**Figure 2:**
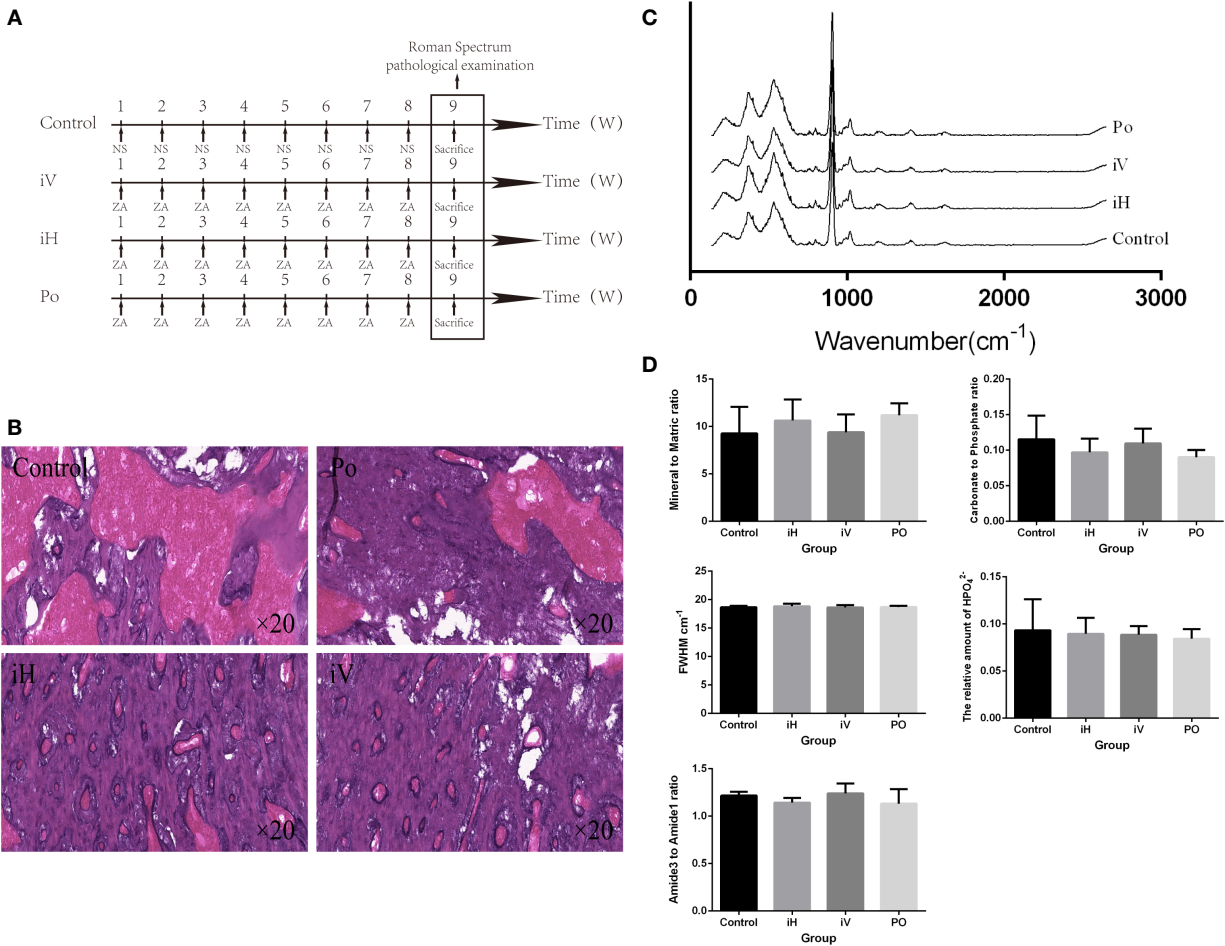
Method-dependent effects of BP administration on the rat mandible. **(A)** Schedule of BP administration to rats. **(B)** Pathology images show increased trabeculae in the cancellous bone region with the BP administration compared with the control group. **(C, D)** The Raman spectra from a different group and the derived parameters show no significant difference in mandibular components and structure (iH: subcutaneous injection, iV: intravenous injection, and PO: oral administration).

Previous studies showed that the risk of BRONJ is higher in patients taking intravenous BPs compared with oral BPs (18). Twelve (12) rats were prospectively and randomly divided into 4 groups to compare the effects of different administration methods on the mandible. Rats in the experimental group were given ZA (100 μg/kg) by tail vein injection, gavage, and local submucosal injection, respectively, every week (**Figure 2A**). On week 9, all rats were sacrificed, and their mandibles were dissected for Raman spectroscopy and pathological examination.

### 2.4 The Raman spectral difference between the lesion and normal bone in the BRONJ mandible

To explore the feasibility of discriminating BRONJ lesion bone with Raman spectroscopy, BRONJ rats model were adopted. Eight (8) rats were given ZA (100 μg/kg) by tail vein injection every week for four (4) weeks. On week 5, surgical stimulation was performed in the rat’s right mandible. Specifically, after removing the hair in the right mandible, the skin was disinfected with 75% ethyl alcohol. Then, a 1 cm incision was made along the lower margin of the mandible to the bone surface. The bone tissues below the molars were ground by slow ball drilling with ice water to the marrow cavity. Finally, the muscle and skin were sutured (17). The rats were fed for another 4–6 weeks until BRONJ symptoms developed. Then, these rats were sent for Micro-CT imaging to confirm the successfully constructed rat BRONJ model. Additionally, the lesion bone tissues acquired from the right mandible and the healthy bone acquired from the left mandible were dissected for Raman spectroscopy examination. Finally, all bone samples were sent for further pathological examination to further validate. BRONJ lesion bone was characterized by necrotic bony trabeculae demonstrating empty osteocyte lacunae in the pathological images.

### 2.5 Raman spectroscopy examination

Following soft tissue removal, the bone tissues below the molars were cut using dental drillers under constant irrigation with distilled water. Slices were then placed on a microscope slide to acquire spectra.

A portable Raman spectrometer (ATR3110, Opus Tiancheng Optoelectronics co. LTD, China) detected all the bone samples with a laser (300 mW; 785 nm). Data were recorded between 200–2600 cm^-1^ at a resolution of approximately 6 cm^-1^. Five (5) spectra were recorded from random sites on each sample. Each spectrum resulted from 2 accumulations, each with a 10 s exposure time. The spectra from each bone sample were averaged to give representative spectra for that bone. Cosmic rays, noise, and the underlying background signal were removed from each spectra using OriginPro 2017 SR2 software. Average spectra were calculated for each bone type by summing all the spectra and dividing by the number of spectra for that group of bone. Spectra were then analyzed in 2 different ways: by directly comparing Raman bands from different groups (**Table 1**) and by comparison of parameters derived from the Raman spectrum from different groups as follows (12, 19, 20):

- Mineral to Matric ratio: calculated by dividing the value from the intensity of Phosphate ν_4_ (mineral) peak (589 cm^-1^) by Amide III (matric) peak (~1260 cm^-1^), which reflects the mineralization of the bone;
- Carbonate to Phosphate ratio: calculated by dividing the value from the carbonate peak (1070 cm^−1^) by the phosphate ν_4_ peak;
- Full Width at Half Maximum Height of Phosphateν_1_ peak (961 cm^-1^) (FWHM): this value, together with the carbonate to phosphate ratio, reflects the degree of crystallinity of the mineral part of the bone;
- The relative amount of HPO_4_^2-^: calculated by dividing the value from the HPO_4_^2-^ (1003 cm^-1^) by the phosphateν_4_ peak;
- Amide III to Amide I ratio: calculated by dividing the value from the Amide III peak by the Amide I peak (~1680 cm^-1^), which reflects the abundance of two kinds of structure within the collagen matrix.

**Table 1:** Raman bands for the bone tissues.

Raman bands	Wavenumber	Annotation
Phosphate ν_2_	438 cm^-1^	Phosphate-bending vibration
Phosphate ν_4_	589 cm^-1^	Phosphate-bending vibration
Phosphate ν_1_	961 cm^-1^	Phosphate symmetric stretching vibration
HPO_4_^2-^	1003 cm^-1^	The relative amount of HPO_4_^2-^
Carν_1_/Phosphateν_3_	1070 cm^-1^	The superposition of carbonate and phosphate v_3_
Amide III	1245–1270 cm^-1^	The relative amount of collagenous organic material
CH2	1445 cm^-1^	The relative amount of both collagenous and non-collagenous organic material
Amide I	1665 cm^-1^	The relative amount of collagenous organic material

### 2.6 Micro-CT examinations

A small animal micro-CT (Hiscan XM, Suzhou Heisfeld information technology co. LTD, China) was used for all CT imaging. Micro-CT scan parameters were as follows: Power: 8 W, Voltage: 60 V, Electric current: 133.3 μA, Detector mode: binging 2×2, Slice thickness: 50 μm, and Repetition time: 75 ms. The CT imaging was processed by SeProcessPro Version.1 software (Version 1.0, Suzhou Heisfeld information technology co. LTD, China).

### 2.7 Pathological examination

Bone samples were decalcified in EDTA (pH 7.20) at 4°C for 21 days. Decalcified samples were embedded in paraffin using the standard method. Then, a series of 4 μm sections were prepared. A professional pathologist observed Hematoxylin-Eosin (HE) stained sections using a light microscope (Nikon H550S, Tokyo, Japan).

### 2.8 Statistical analysis

Statistical analysis was performed using SPSS statistical software (version 23.0, IBM, Chicago, Illinois, USA). Values are presented as the mean ± standard deviation. Mean values from each parameter were compared using 2-way ANOVA with times and modes of BP administration as the two independent variables. The Student’s t-test was used to compare the parameter difference between the affected and healthy bone of the mandible. Raman bands and parameters derived from Raman spectra were presented for principal component analysis. *P* < 0.05 was considered statistically significant.

## 3 Results

### 3.1 Time-dependent effects of BP administration on the rat mandible

After different BP administration times, all right mandibles were dissected for further Raman spectroscopy examination and pathological examination. The averaged Raman spectra of the different groups are shown in **Figure 1C**. No difference could be seen between these groups in the Raman spectrum and the parameters derived (**Figure 1D**). However, the pathology images show that, with the increase of BP administration times, there are increased trabeculae in the cancellous bone region (**Figure 1B**).

### 3.2 Mode-dependent effects of BP administration on the rat mandible

All right mandibles were dissected for further Raman spectroscopy examination and pathological examination after the BPs were administered differently to the rats. The averaged Raman spectra of the different groups (**Figure 2C**). No difference could be seen between these groups in the Raman spectral and the parameters derived (**Figure 2D**). But compared with the control group, trabeculae in the cancellous bone region increased in the iV, iH, and PO groups (**Figure 2B**), and trabeculae in the cancellous bone region increased the most in the iV group.

### 3.3 The Raman spectra difference between the lesion and normal bone in the BRONJ mandible

**Figure 3:**
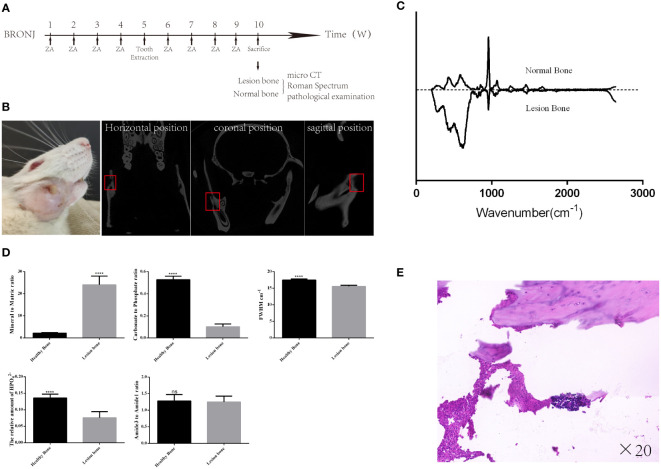
The Raman spectra difference between the lesion and normal bone in the BRONJ mandible. **(A)** Schedule of the rat BRONJ model construction. **(B)** The camera and micro-CT image of the rat with BRONJ. **(C)** The Raman spectrum from healthy and lesion bone in the rat mandible with BRONJ shows significant differences. **(D)** Various parameters derived from Raman spectra show obvious changes in mandibular components and structure. **(E)** The pathological image shows the formation of dead bone and infiltration of inflammatory cells, further confirming the successful construction of the BRONJ model of rat. **** = P<0.0001.

BRONJ was successfully constructed in six (6) out of the eight (8) rats tested (**Figure 3A, B**, **E**). All mandibles, including the lesion and healthy bones, were analyzed by Raman spectroscopy. The average Raman spectra are shown in **Figure 3C**. The Mineral to Matric ratio showed significant increases in lesion bone compared with healthy bone (Mineral to Matric ratio: from 2.22 ± 0.09 to 24.06 ± 1.61, P<0.0001) (**Figure 3D**). While Carbonate to Phosphate ratio, the relative amount of HPO4^2^, and FWHM had significant decreases in lesion bone compared with healthy bone (Carbonate to Phosphate ratio: from 0.528 ± 0.013 to 0.102 ± 0.010, P<0.001, the relative amount of HPO4^2-^: from 0.136 ± 0.005 to 0.076 ± 0.008, P<0.0001, FWHM: from 17.5 ± 0.124 to 15.58 ± 0.126, P<0.0001)

### 3.4 PCA analysis

**Figure 4:**
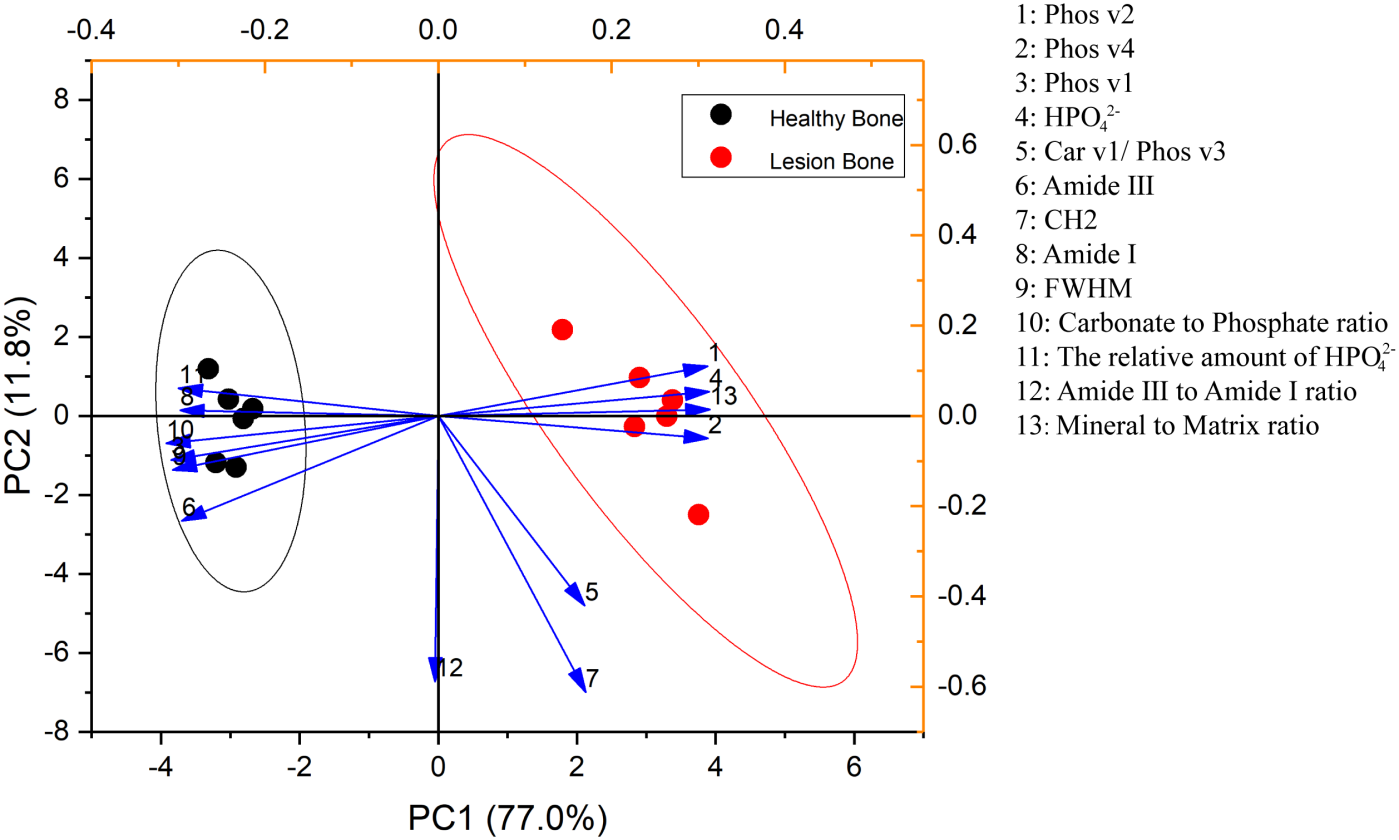
PCA analysis comparing the lesion bone of BRONJ and healthy bone.

The first two principal component loadings describe the primary information obtained from the PCA: PC1 and PC2 (**Figure 4**). Among them, PC1 accounted for 77.0% of the variance within the data, which reflects the changed mandible bone tissue composition, and PC2 accounted for 11.8% of the variance, which reflects the unchanged mandible bone tissue composition. The scores from PC1 from lesion bone of BRONJ and healthy bone were significantly different (-2.99 ± 0.98 to 2.99 ± 0.27, P<0.01), but not in PC2 (-0.13 ± 0.39 to 0.13 ± 0.63, P= 0.74).

## 4 Discussion

In previous studies, different animals were chosen to mimic the disease progression of BRONJ. For instance, Burr DB (21) and Pautke C (22) et al. successfully constructed the BRONJ animal model using beagles and miniature pigs after long-term administration of BPs, but these methods were not widely adopted because of the high cost. Compared with large animals, there are several advantages to utilizing SD rats to build the BRONJ model (23). First, SD rats are mammals, and the completed gene map shows similarities between rats and human genes. Second, resulting from the large size of its jaw bone, it is convenient for experimental operation. Third, SD rats breed rapidly and are easy to feed, allowing for cheaper experiments. In this study, SD rats were selected to construct the BRONJ model.

After reviewing previous studies, BRONJ animal models were all constructed based on repeated administration of BPs and traumatic stimulation such as tooth extraction and local surgery (17, 23–25). According to previous literature, Bisphosphonates (P-C-P) are chemically stable derivatives of pyrophosphate (P-O-P), with the oxygen being replaced by carbon that renders protection against breakdown by hydrolysis and mediates a tight binding to the hydroxyapatite crystals in the bone (26). In this study, we first explored the time- and method-dependent effects of BP administration on the rat mandible with the help of Raman spectroscopy and pathological examination. However, no obvious difference was found from the Raman spectra which aroused our suspicions. So the bone tissues were further sent to perform pathological examination image, and the results showed increased trabeculae in the cancellous bone region with increased BP administration times. This means the bone tissue was indeed affected by BPs administration. Through further investigating the previous literatures, we found BPs would reduce the absorption of bone tissues and disrupt the balance of bone remodeling by inhibiting osteoclast activity (26). In other words, it does not affect the composition of the mandible when only BPs is administered. This also means local stimulation may play a more important role in the genesis of BRONJ. Moreover, trabeculae increased the most using intravenous administration, compared with a local injection or oral administration. Previous studies have reported that when administered intravenously, BP is loaded into the bone and accumulates 142.8 times faster than when administered orally (27). Therefore, it can be concluded that there is a positive correlation between the changes in mandibular structure and the BP dose administered. But only with the administration of BPs, no rats showed symptoms of BRONJ. Next, we began to build the SD rat model of BRONJ with repeated intravenous administration of BPs and local stimulation by grinding the mandible. Finally, six (6/8) rats showed symptoms of BRONJ. This means that BP administration and local stimulation play an essential role in BRONJ development. Lastly, BRONJ can be prevented by reducing trauma, infection, and other local stimulation as well.

Currently, conservative and surgical treatment regimens are recommended (2–5, 10). However, the more knowledge we gain regarding BRONJ, the more surgical therapy is recommended because the success rates are higher, the progression of the disease can be controlled, and the diagnosis of osteonecrosis can be proved histologically (28). It is difficult for surgeons to accurately decide the boundaries of lesion bones that will allow for the balance of preserving healthy bone and removing the lesion bone completely (16, 29–31). Therefore, it is significant to establish an appropriate method to distinguish the boundaries between the lesion and healthy bone accurately. In this study, both lesions and healthy bone were detected by Raman spectroscopy. The Raman spectrum shows a significant difference between the lesion and healthy bone. Therefore, during the surgery, the surgeon could utilize Raman spectroscopy to determine the surgical boundary before removing BRONJ lesion bone tissue. Moreover, Raman spectroscopy could also be used to monitor the surgical wound after removing BRONJ lesion bone to ensure the completely removal of lesion bone. That means it may be a novel, feasible method for surgeons to accurately remove lesion bone in a patient with BRONJ utilizing the Raman spectrum.

## Data availability statement

The raw data supporting the conclusions of this article will be made available by the authors, without undue reservation.

## Ethics statement

The animal study was reviewed and approved by the Animal Ethics Committee of the Institute Affiliated Stomatology Hospital, Nanjing University Medical School.

## Author contributions

CX performed the experiments, edited the manuscript, and performed data curation, analysis, and visualization. YP contributed to the experiments and data analysis, and QZ contributed to the experiments and data analysis. QH provided funding and supervision and reviewed the manuscript, and YW contributed to the design and methodology and revised the manuscript. All authors contributed to the article and approved the submitted version.

## References

[1] McClung M Harris ST Miller PD Bauer DC Davison KS Dian L. Bisphosphonate therapy for osteoporosis: benefits, risks, and drug holiday. Am J Med (2013) 126(1):13–20. doi: 10.1016/j.amjmed.2012.06.023 23177553

[2] Sun J Wen X Jin F Li Y Hu J Sun Y. Bioinformatics analyses of differentially expressed genes associated with bisphosphonate-related osteonecrosis of the jaw in patients with multiple myeloma. Onco Targets Ther (2015) 8:2681–8. doi: 10.2147/OTT.S88463 PMC4590669 26445550

[3] Dudek NL Croft NP Schittenhelm RB Ramarathinam SH Purcell AW. A systems approach to understand antigen presentation and the immune response. Methods Mol Biol (2016) 1394:189–209. doi: 10.1007/978-1-4939-3341-9_14 26700050

[4] Cella L Oppici A Arbasi M Moretto M Piepoli MF Vallisa D. Autologous bone marrow stem cell intralesional transplantation repairing bisphosphonate related osteonecrosis of the jaw. Head Face Med (2011) 7(1):16. doi: 10.1186/1746-160X-7-16 21849044 PMC3175443

[5] Curi MM Cossolin GS Koga DH Zardetto C Christianini S Feher O. Bisphosphonate-related osteonecrosis of the jaws–an initial case series report of treatment combining partial bone resection and autologous platelet-rich plasma. J Oral Maxillofac Surg (2011) 69(9):2465–72. doi: 10.1016/j.joms.2011.02.078 21763050

[6] Rugani P Luschin G Jakse N Kirnbauer B Lang U Acham S. Prevalence of bisphosphonate-associated osteonecrosis of the jaw after intravenous zoledronate infusions in patients with early breast cancer. Clin Oral Investig (2014) 18(2):401–7. doi: 10.1007/s00784-013-1012-5 23749244

[7] Migliorati CA Epstein JB Abt E Berenson JR. Osteonecrosis of the jaw and bisphosphonates in cancer: a narrative review. Nat Rev Endocrinol (2011) 7(1):34–42. doi: 10.1038/nrendo.2010.195 21079615

[8] McDonald MM Dulai S Godfrey C Amanat N Sztynda T Little DG. Bolus or weekly zoledronic acid administration does not delay endochondral fracture repair but weekly dosing enhances delays in hard callus remodeling. Bone. (2008) 43(4):653–62. doi: 10.1016/j.bone.2008.05.019 18582604

[9] Walter C Pabst A Ziebart T Klein M Al-Nawas B. Bisphosphonates affect migration ability and cell viability of HUVEC, fibroblasts and osteoblasts in vitro. Oral Dis (2011) 17(2):194–9. doi: 10.1111/j.1601-0825.2010.01720.x 20796232

[10] Boff RC Salum FG Figueiredo MA Cherubini K. Important aspects regarding the role of microorganisms in bisphosphonate-related osteonecrosis of the jaws. Arch Oral Biol (2014) 59(8):790–9. doi: 10.1016/j.archoralbio.2014.05.002 24859766

[11] Lesclous P Grabar S Abi Najm S Carrel JP Lombardi T Saffar JL. Relevance of surgical management of patients affected by bisphosphonate-associated osteonecrosis of the jaws. A prospective Clin radiological study Clin Oral Investig (2014) 18(2):391–9. doi: 10.1007/s00784-013-0979-2 23604698

[12] Goodyear SR Gibson IR Skakle JM Wells RP Aspden RM. A comparison of cortical and trabecular bone from C57 black 6 mice using raman spectroscopy. Bone. (2009) 44(5):899–907. doi: 10.1016/j.bone.2009.01.008 19284975

[13] Caraher MC Sophocleous A Beattie JR O'Driscoll O Cummins NM Brennan O. Raman spectroscopy predicts the link between claw keratin and bone collagen structure in a rodent model of oestrogen deficiency. Biochim Biophys Acta Mol Basis Dis (2018) 1864(2):398–406. doi: 10.1016/j.bbadis.2017.10.020 29066282

[14] Khalid M Bora T Ghaithi AA Thukral S Dutta J. Raman spectroscopy detects changes in bone mineral quality and collagen cross-linkage in staphylococcus infected human bone. Sci Rep (2018) 8(1):9417. doi: 10.1038/s41598-018-27752-z 29925892 PMC6010429

[15] Mannelli G Comini LV Piazza C. Surgical margins in oral squamous cell cancer : intraoperative evaluation and prognostic impact. Curr Opin Otolaryngol Head Neck Surgery (2019) 27(2):98–103. doi: 10.1097/MOO.0000000000000516 30844923

[16] Ristow O Otto S Geiss C Kehl V Berger M Troeltzsch M. Comparison of auto-fluorescence and tetracycline fluorescence for guided bone surgery of medication-related osteonecrosis of the jaw: a randomized controlled feasibility study. Int J Oral Maxillofac Surg (2017) 46(2):157–66. doi: 10.1016/j.ijom.2016.10.008 27856150

[17] Tsurushima H Kokuryo S Sakaguchi O Tanaka J Tominaga K. Bacterial promotion of bisphosphonate-induced osteonecrosis in wistar rats. Int J Oral Maxillofac Surg (2013) 42(11):1481–7. doi: 10.1016/j.ijom.2013.06.011 23932020

[18] Yamazaki T Takahashi K Bessho K. Recent clinical evidence in bisphosphonate-related osteomyelitis of the jaw: focus on risk, prevention and treatment. Rev Recent Clin Trials (2014) 9(1):37–52. doi: 10.2174/1574887109666140423120614 24758539

[19] Kun-Darbois JD Libouban H Mabilleau G Pascaretti-Grizon F Chappard D. Bone mineralization and vascularization in bisphosphonate-related osteonecrosis of the jaw: an experimental study in the rat. Clin Oral Investig (2018) 22(9):2997–3006. doi: 10.1007/s00784-018-2385-2 29453497

[20] Gamsjaeger S Masic A Roschger P Kazanci M Dunlop JW Klaushofer K. Cortical bone composition and orientation as a function of animal and tissue age in mice by raman spectroscopy. Bone. (2010) 47(2):392–9. doi: 10.1016/j.bone.2010.04.608 20450992

[21] Burr DB Liu Z Allen MR. Duration-dependent effects of clinically relevant oral alendronate doses on cortical bone toughness in beagle dogs. Bone. (2015) 71:58–62. doi: 10.1016/j.bone.2014.10.010 25445446 PMC4274196

[22] Pautke C Bauer F Bissinger O Tischer T Kreutzer K Steiner T. Tetracycline bone fluorescence: a valuable marker for osteonecrosis characterization and therapy. J Oral Maxillofac Surg (2010) 68(1):125–9. doi: 10.1016/j.joms.2009.05.442 20006166

[23] Yang H Pan H Yu F Chen K Shang G Xu Y. A novel model of bisphosphonate-related osteonecrosis of the jaw in rats. Int J Clin Exp Pathology (2015) 8(5):5161–7. PMC4503084 26191212

[24] Lopez-Jornet P Camacho-Alonso F Molina-Minano F Gomez-Garcia F Vicente-Ortega V. An experimental study of bisphosphonate-induced jaws osteonecrosis in sprague-dawley rats. J Oral Pathol Med (2010) 39(9):697–702. doi: 10.1111/j.1600-0714.2010.00927.x 20819131

[25] Silva PG Ferreira Junior AE Teofilo CR Barbosa MC Lima Junior RC Sousa FB. Effect of different doses of zoledronic acid in establishing of bisphosphonate-related osteonecrosis. Arch Oral Biol (2015) 60(9):1237–45. doi: 10.1016/j.archoralbio.2015.05.015 26093347

[26] De Ceulaer J Tacconelli E Vandecasteele SJ. Actinomyces osteomyelitis in bisphosphonate-related osteonecrosis of the jaw (BRONJ): the missing link? Eur J Clin Microbiol Infect Dis (2014) 33(11):1873–80. doi: 10.1007/s10096-014-2160-5 24880820

[27] Marx RE. A decade of bisphosphonate bone complications: what it has taught us about bone physiology. Int J Oral Maxillofac Implants (2014) 29(2):e247–58. doi: 10.11607/jomi.te61 24683588

[28] Dayisoylu EH Ungor C Tosun E Ersoz S Kadioglu Duman M Taskesen F. Does an alkaline environment prevent the development of bisphosphonate-related osteonecrosis of the jaw? an experimental study in rats. Oral Surg Oral Med Oral Pathol Oral Radiol (2014) 117(3):329–34. doi: 10.1016/j.oooo.2013.11.490 24368141

[29] Elad S Gomori MJ Ben-Ami N Friedlander-Barenboim S Regev E Lazarovici TS. Bisphosphonate-related osteonecrosis of the jaw: clinical correlations with computerized tomography presentation. Clin Oral Investig (2010) 14(1):43–50. doi: 10.1007/s00784-009-0311-3 19603201

[30] Wilde F Steinhoff K Frerich B Schulz T Winter K Hemprich A. Positron-emission tomography imaging in the diagnosis of bisphosphonate-related osteonecrosis of the jaw. Oral Surg Oral Med Oral Pathol Oral Radiol Endod (2009) 107(3):412–9. doi: 10.1016/j.tripleo.2008.09.019 19121962

[31] Otto S Pache C Mast G Ehrenfeld M Pautke C. Outcome evaluation of fluorescence-guided bone resection for the treatment of bisphosphonate-related osteonecrosis of the jaw. Int J Oral Maxillofac Surgery (2013) 42(10):1189–90. doi: 10.1016/j.ijom.2013.07.075

